# UK animal therapists’ confidence in executing professional skills in clinical practice

**DOI:** 10.1002/vetr.4704

**Published:** 2024-11-17

**Authors:** Alice Sear, Alison P. Wills

**Affiliations:** ^1^ Department of Animal and Agriculture Hartpury University Gloucester UK

## Abstract

**Background:**

Veterinary physiotherapy and hydrotherapy are popular modalities in the UK, yet little is known about the confidence of therapists responsible for performing these treatments. A lack of confidence may have a detrimental impact on patients; therefore, this study investigated the effect of age, species specialisation, educational level, occupation, qualifications, experience and years practising on animal therapists' confidence in executing professional skills.

**Methods:**

An online survey was conducted to collect quantitative data regarding UK‐based therapists’ confidence in the completion of entrustable professional activities (EPAs). In addition, semi‐structured interviews were conducted to explore participants' perceptions of therapy qualifications and their personal experiences in clinical practice.

**Results:**

Occupation, educational level and therapy qualification had a significant effect (*p* < 0.05) on participants' confidence in performing all EPAs. Experience had a significant effect (*p* < 0.05) on four EPAs; those with less than 1 year of experience had the lowest confidence scores, with increased experience in the animal industry and as an animal therapist resulting in a rise in confidence.

**Limitations:**

This study relied on participants reporting their own limitations and could have further explored why animal therapists choose to obtain specific qualifications.

**Conclusions:**

Providing support to new graduates may improve their confidence. In addition, it is important that qualifications adequately prepare animal therapists for clinical practice.

## INTRODUCTION

Hydrotherapy and veterinary physiotherapy have increased in popularity in the UK over recent years, primarily for the rehabilitation of acute and chronic conditions in dogs and for optimising performance in horses.^1^, ^2^, ^3^ They have been shown to be effective in increasing the range of motion in the distal limb,^4^, ^5^ in addition to increasing muscle mass, which often becomes depleted following periods of rest after surgery.^2^, ^6^, ^7^, ^8^ These findings indicate that providing physiotherapy and hydrotherapy is effective in promoting recovery and performance in dogs and horses, with awareness of these modalities demonstrated by some equine veterinarians.^9^ In a survey‐based study of 161 veterinarians, 53.6% outlined that they would consider referring patients for hydrotherapy following a cranial cruciate ligament rupture, and 41.9% reported that they would consider physiotherapy.^10^ Despite this, 96.0% (*n* = 74) of veterinarians responding to a similar survey suggested that more research needs to be published on the effects of physiotherapy.^11^ Similarly, some doctors working in human healthcare and various veterinary organisations have expressed concerns over unknown outcomes following the use of complementary and alternative medicines.^12^, ^13^, ^14^ Over 10 years ago, almost 50% of staff working in hydrotherapy centres across the UK were found not to have a nationally recognised qualification.^1^ Furthermore, some veterinarians have expressed concerns that not all persons involved in performing alternative treatments have the appropriate experience or knowledge to provide animals with a quality treatment programme.^9^, ^11^, ^15^

While the amount of training between and within veterinary physiotherapy and animal hydrotherapy qualifications does vary, many courses in the UK are accredited by governing bodies that promote high professional standards and competency.^3^, ^16^, ^17^, ^18^ However, there are various governing bodies for veterinary physiotherapy and hydrotherapy in the UK, with courses provided by both universities and other providers, meaning that course content may vary. Competence is essential for ensuring that both human and animal patients remain safe within clinical therapy practice; however, research from other disciplines suggests that confidence may be equally meaningful.^19^, ^20^

Confidence is defined as the belief or assurance in one's self, while professional confidence relates specifically to an individual's inner self‐confidence when completing work‐related tasks.^19^, ^21^, ^22^ Medical nurses who lack confidence in their abilities may be unable to provide sufficient standards of care, while surgeons could be vulnerable to bouts of indecision and doubt, both of which can be dangerous for patients.^19^, ^20^ Conversely, while female nursing students often outline lower levels of confidence compared to their male counterparts,^23^ competence does not differ.^24^ This indicates that lower confidence does not always impact competence in all clinical skills. Instead, specific competencies, such as verbal communication, or practical skills, including prescribing drugs or performing surgical procedures, may be impacted.^19^, ^24^, ^25^ This has not been explored in animal therapists performing alternative and complementary therapies, who are often required to undertake practical and communicative tasks daily.

Currently, confidence levels among animal therapists are unknown; however, experience and exposure to clinical scenarios have been outlined in the medical and veterinary literature as significant factors affecting confidence,^23^, ^25^, ^26^ and these may apply similarly to animal therapists. For example, experienced nurses ranked themselves as significantly more confident in executing high‐fidelity simulation training exercises compared to their student nurse counterparts; however, this was not positively correlated with increased competence.^26^ Fourth‐year nursing students reported significantly lower self‐confidence compared to second‐year nursing students, indicating that confidence may not increase linearly throughout students’ time at university.^23^ Fluctuations in confidence often occurred among newly graduated nurses, with confidence being highly dependent on the task they were required to execute and whether mistakes were made throughout the day.^25^ It is likely that this phenomenon may also exist among newly qualified veterinary paraprofessionals, where some skills may become easier with time. However, other skills may remain challenging throughout some professionals’ careers regardless of experience.^27^, ^28^

This study aimed to determine whether age, species specialisation, educational level, occupation, qualifications, experience, and years practising as an animal therapist impacted the confidence of UK veterinary physiotherapists and animal hydrotherapists in executing entrustable professional activities (EPAs). EPAs are a set of tasks that typically aim to evaluate competence in a range of professions, including among veterinarians and doctors.^29^, ^30^, ^31^

## METHODS

Ethical approval for this project was granted by the Hartpury University Ethics Committee on 23 March 2022 (ETHICS2021‐78). This project followed a cross‐sectional design to gain a range of opinions from animal hydrotherapists and veterinary physiotherapists across the UK. Quantitative and qualitative data were collected; quantitative data from an online survey and qualitative data from follow‐up interviews.

### Questionnaire design

An online questionnaire was created in Microsoft Forms. Section one consisted of closed questions to gather categorical data on participant demographics, including age, sex and species treated.^22^, ^32^, ^33^ Participants were asked for their occupation and were able to select from veterinary physiotherapist, veterinary physiotherapist and hydrotherapist, equine hydrotherapist and canine hydrotherapist. Section two aimed to determine participants’ confidence in completing specific EPAs using a series of five‐point Likert scales with an additional category for not applicable. Some EPAs were based on previous literature^30^, ^34^ while others were created to ensure that they remained specific and relevant to animal hydrotherapists and veterinary physiotherapists, such as keeping accurate records of each treatment session. Each EPA was clearly defined based on published guidance from the RCVS.^35^ EPAs covered topics such as interpreting veterinary referrals, performing clinical assessments, constructing treatment plans, performing risk assessments and communication skills. An average EPA score was also calculated for each participant. The final section included a space for participants to leave an email address if they wished to be contacted to participate in a follow‐up interview. The full questionnaire can be seen in the online Supporting Information.

### Questionnaire distribution

Surveys were distributed over a 2‐week period and were posted on social media groups and promoted via targeted email groups within the host institution. The participants provided informed consent at the start of the survey.

### Questionnaire analysis

Participants were assigned numbers for anonymity and variables were combined when the total count for nominal variables was less than five responses. Since the independent variables were nominal, a non‐normal distribution was assumed. Kruskal‒Wallis tests were conducted to explore the effect of age, occupation, level of qualification, educational level and experience on both average and individual EPA scores. Post hoc tests with Bonferroni correction were performed where significant main effects were identified. Fisher's exact test was used to test for an association between age and occupation. Mann‒Whitney tests were used to test for the effect of species specialisation (companion animal or equine) on both average and individual EPA scores. A *p*‐value of less than 0.05 was deemed statistically significant for all tests. Tests were not performed to assess the effect of sex or type of working environment on EPA scores due to the small numbers in some groups precluding valid statistical analysis.

### Interview design

Qualitative data were collected to provide a more in‐depth perspective of the confidence of veterinary paraprofessionals in completing EPAs. Individual interview questions aimed to explore participants’ experiences of their therapy qualifications,^36^ and how their education impacted their confidence in completing specific EPAs.^37^

### Interview protocol

Interviews took place over a 2‐week period. Consent was acquired via email prior to the one‐to‐one structured interviews, which took place online via Microsoft Teams. The purpose of the interview was explained to each participant as part of an information document, in addition to a verbal briefing before the interview began. The participants were asked not to mention any businesses, colleagues or employees by name to protect their anonymity. The participants were asked each question verbally, and the questions were also simultaneously being provided in written form on a screen. The interview participants (*n* = 4) were anonymised: one was a canine hydrotherapist, one was a veterinary physiotherapist and two were veterinary physiotherapists specialising in canine hydrotherapy.

### Interview analysis

The interviews were audio recorded and automatic transcription was applied. This was used as a basis for verbatim transcription. Qualitative data were not formally analysed due to the small sample size, but qualitative comments made in interviews are reported where relevant to expand on the results of the quantitative analysis.

## RESULTS

The total number of responses for the survey was 79. Of the respondents, 63 (79.7%) treated small animals, while the remaining 16 (20.3%) treated horses. In terms of employment type, nine (11.4%) worked in a primary care or referral veterinary practice, one (1.3%) was located in a research or education‐based facility and 69 (87.3%) worked in independent businesses. Most respondents (97.5%; *n* = 77) were female. The majority (*n* = 31; 39.2%) were aged between 21 and 30 years, 19 (24.1%) were aged between 31 and 40 years, 20 (25.3%) were aged between 41 and 50 years and nine (11.4%) were aged 51 years or over.

### Age

There was no significant effect of age on any of the individual EPAs or average EPA score (*p* > 0.05). There was no significant association between occupation and age (*p* = 0.128).

### Species specialisation

There was no significant effect of species specialisation (equine or companion animal) on any of the individual EPAs or average EPA score (*p* > 0.05). Interview data revealed that three of the four participants felt more confident in treating dogs, which was consistent with their current species specialisation. One interviewee highlighted that when they graduated they were equally confident in treating dogs and horses, but their recent specialisation in canine therapy meant that they are ‘a bit out of practice with horses’ now.

### Educational level

There was no significant main effect of educational level on average EPA scores (*H*(2) = 1.912, *p* = 0.385). However, there was a significant main effect of educational level on the individual EPA regarding the ability to develop and implement a management or treatment plan for a patient (*H*(2) = 9.316, *p* = 0.009). Individuals with a level seven (postgraduate degree) qualification or above rated their ability higher (mean score = 45.38) than those that had a level six qualification (undergraduate degree) (mean score = 33.11; *p* = 0.04); however, those that had a level one, two or three qualification (GCSEs and/or A‐levels) also rated their ability higher (mean score = 47.00) than those with a level six qualification (*p* = 0.03). There was no significant difference between those who had a level one, two or three qualification and those who held a level seven qualification or above (*p* = 1.00). Some of the interview participants held a level seven qualification, and one highlighted that ‘you come out of a Master's and you [think you] know everything, you don't’. Another interviewee commented ‘I would recommend taking on a higher‐level qualification first and then specialising in hydrotherapy’.

### Occupation

There was no significant main effect of occupation on average EPA scores (*H*(2) = 1.501, *p* = 0.47). However, when looking at individual EPAs, there was a statistically significant effect of occupation on therapists’ ability to keep accurate treatment records (*H*(2) = 12.986, *p* = 0.002), with canine hydrotherapists rating their ability significantly higher (mean score = 42.84) than veterinary physiotherapists (mean score = 30.72; *p* = 0.044) and those that were both veterinary physiotherapists and canine hydrotherapists rating themselves higher (mean score = 49.80) than veterinary physiotherapists (*p* = 0.002). The remaining pairwise comparisons were non‐significant (*p* > 0.05). In the interviews, both a canine hydrotherapist and veterinary physiotherapist highlighted the time‐consuming nature of keeping treatment records ‘it really does take the most work for me’ and ‘it can take a while … and being self‐employed’. One interview participant, who was both a veterinary physiotherapist and a canine hydrotherapist, explained the benefit of software they had invested in ‘so there is obviously the software I've invested in … which has made … streamlining everything a lot easier … it's also taken my note time down, which gives me more time to actually spend looking over the animal … rather than having to write stuff down’.

### Therapy qualifications

There was no significant main effect of the type of therapy qualification held on average EPA scores (*H*(4) = 4.401, *p* = 0.354). However, when looking at individual EPAs, there was a significant main effect of type of therapy qualification on confidence interpreting veterinary referrals (*H*(4) = 9.891, *p* = 0.042), with those with a level six diploma in veterinary physiotherapy (mean score = 47.43) having significantly higher confidence than those with a level six Bachelor's degree in veterinary physiotherapy (mean score = 25.00; *p* = 0.033). All other pairwise comparisons were non‐significant (*p* > 0.05). There was also a significant main effect of qualification on respondents' ability to recognise if a patient requires urgent or emergency care (*H*(2) = 9.721, *p* = 0.045). However, no significant differences between individual qualifications were found in the pairwise comparisons (*p* > 0.05). The interview participants highlighted that they rarely experienced emergency situations: ‘I don't tend to get anything like that’, ‘we have had maybe two emergencies in 10 years’. One interviewee, who was a veterinary physiotherapist, mentioned that an emergency occurred, but they were in a veterinary practice at the time ‘luckily I was at the vets … they got … on it immediately’.

### Experience in the animal industry

There was a significant main effect of years spent in the animal industry on overall EPA score (*H*(4) = 11.161, *p* = 0.025). However, no significant differences were found in the pairwise comparisons (*p* > 0.05). In addition, length of time spent in the animal industry had a significant effect on four individual EPAs. There was a significant main effect of experience working in the animal industry on confidence in gathering a clinical history during an initial appointment (*H*(4) = 9.677, *p* = 0.046). Individuals who had 11‒20 years of industry experience had greater confidence (mean score = 46.00) than those who had less than 1 year of experience (mean score = 19.42; *p* = 0.027). There was also a significant main effect of experience in the animal industry on confidence in interpreting a veterinary referral (*H*(4) = 10.829, *p* = 0.029). Compared with those who had less than 1 year of experience (mean score = 18.50), those who had 11‒20 years (mean score = 44.64; *p* = 0.027) or 21 years plus experience (mean score = 45.00; *p* = 0.026) in the industry were significantly more confident. There was also a significant main effect of experience in the animal industry on confidence in interpreting a clinical history (*H*(4) = 9.59, *p* = 0.048). However, no significant differences between experience categories (in years) were found in the pairwise comparisons (*p* > 0.05). There was a significant main effect of experience in the animal industry on confidence in recognising a patient that requires urgent or emergency care (*H*(4) = 19.099; *p* = 0.001). Compared with those who had worked in the industry for less than 1 year (mean score = 19.33), those who had worked in the industry for 1‒5 years (mean score = 47.00; *p* = 0.010), 11‒20 years (mean score = 45.57; *p* = 0.027) or 21 years or more (mean score = 48.08; *p* = 0.012) all had significantly greater confidence.

The interview participants predominantly had experience in their respective fields as veterinary physiotherapists and/or canine hydrotherapists, with limited mention of wider experience in the animal industry. However, one participant expressed an interest in gaining knowledge in animal nutrition and training and suggested that they might employ staff with this expertise: ‘I can help more dogs by gathering the right people’. All of the interviewees mentioned continued professional development (CPD) as a means of broadening their knowledge and experience. Some highlighted the requirement of their governing bodies ‘I'm part of two professional bodies so I have to do … [CPD] each year for that’, in addition to mentioning barriers to CPD such as the cost and availability ‘there doesn't seem to be a lot of canine hydrotherapy CPD courses available’, ‘very expensive … being self‐employed’. One participant, who was a canine hydrotherapist, mentioned that note taking was covered by CPD ‘CPD days … helping you develop clinical skills … skills in writing notes and things like that as well, so in vet reporting’.

### Years practising as an animal therapist

There was no significant main effect of length of time practising as an animal therapist on average EPA scores (*H*(3) = 3.284; *p* = 0.350). There was a significant main effect of years spent as an animal therapist on confidence in interpreting a veterinary referral (*H*(3) = 8.186; *p* = 0.042). However, no significant differences were found in the pairwise comparisons (*p* > 0.05). There was also a significant main effect of time spent as an animal therapist on confidence in recognising a patient that requires urgent or emergency care (*H*(3) = 14.794; *p* = 0.002). Those who had been in the role for under a year (mean score = 23.36) were significantly less confident than those who had been in the role for 1‒5 years (mean score = 45.93; *p* = 0.002) or more than 11 years (mean score = 45.57; *p* = 0.012). The interview participants highlighted the challenge of interpreting a veterinary referral when they began their career and how this improved over time. One individual who was both a veterinary physiotherapist and hydrotherapist stated ‘particularly the interpreting the referral was quite difficult … I don't get a lot of information on my actual referral form’, ‘It took me a while to sort of build my confidence in that’. A canine hydrotherapist stated ‘interpreting a veterinary referral, there was a lot of Google … when I first started, figuring out what different veterinary terms mean’, ‘ten years on, still figuring some things out … but pretty good now’. Another interview participant, who was both a veterinary physiotherapist and a canine hydrotherapist, mentioned ‘when I first started … I would say my confidence was kind of like 50/50 quite low … every time I had a vet referral form, I had to go in, research it’.

## DISCUSSION

This study identified a number of factors that significantly affected therapists’ confidence in completing a range of EPAs. Neither the age of the survey respondents nor their species specialisation affected confidence in any of the EPAs.

### The effect of educational level

Educational level affected the EPA regarding the ability to develop and implement a treatment or management plan. Individuals with a level six qualification were least confident in this skill, below those with a level seven qualification and those with a level one, two or three qualification. This finding may be related to specific therapy qualifications that are level six rather than level seven; however, this EPA was not affected by the specific therapy qualification held. Respondents were asked to state their highest educational level, which was not necessarily their therapy qualification (e.g., they could hold a higher‐level qualification in a different subject). The fact that having a postgraduate qualification increases this EPA is unsurprising as the transferable skills developed could help with devising appropriate treatment plans^38^; however, the reason for the high confidence among those with a lower level of education is unclear. These individuals may have gained experience through practical learning and their confidence is independent of their academic qualifications.^26^

### The effect of occupation on confidence

Occupation, which differentiated the roles of veterinary paraprofessionals into veterinary physiotherapists, canine hydrotherapists and both veterinary physiotherapists and hydrotherapists, did not have a significant impact on average confidence scores. The exception to this was the EPA that investigated therapists’ ability to keep accurate treatment records, where veterinary physiotherapists had the lowest confidence. This finding was unexpected given that hydrotherapists may not hold a nationally recognised qualification,^1^ whereas veterinary physiotherapists often belong to accredited organisations.^15^ The logistics around the ability to maintain records may vary between occupations, as veterinary physiotherapists often travel to treat animal patients within their homes^39^ while canine hydrotherapists may be based within fixed locations such as boarding kennels, rehabilitation centres and pet shops.^1^ Research suggests that the use of mobile devices has become integral within the medical environment to access clinical histories and gather information during patient assessments.^40^, ^41^ During an interview, one participant outlined that the use of software had been a fundamental addition in assisting with keeping treatment records, so this is something that may benefit animal therapists more generally.

### The effect of therapy qualification on confidence

Participants with a level 6 diploma in veterinary physiotherapy reported significantly higher confidence scores when interpreting veterinary referrals than those who had undertaken a Bachelor's degree in veterinary physiotherapy; however, therapy qualification had no significant effect on confidence levels for any other EPAs. Where a patient's veterinarian has referred them for therapy, the therapist needs to be able to understand the veterinary terminology used and have awareness of the condition in order to implement a customised treatment plan. Those completing a level six qualification may enter with an existing qualification and or industry experience, whereas those who have only completed the Bachelor's degree might have less breadth of experience. However, more research into the demographics of degree entrants would be needed to confirm this. The content and teaching styles of the courses may also have differed, particularly in relation to the proportion of time spent on admin‐based skills. Teaching styles are likely to vary between educational institutions, which has been highlighted as a limitation in single‐site education research.^24^, ^42^ Data from the interviews suggested that some individuals of different occupations lacked confidence in interpreting veterinary referrals when they first began their careers. As animal therapists become more experienced in their role and benefit from experiential learning, the specific course or qualification they have completed may have less influence on their clinical practice. However, it remains crucial that new graduates enter the industry with sufficient competence and confidence to safeguard their patients and themselves.^19^, ^20^ Research has found that veterinary physiotherapists who were members of governing bodies were slightly more likely to use outcome measures^43^; hence, making membership a requirement might increase confidence and competence in all areas of practice.

### The effect of experience in the animal industry on confidence

Participants with less experience in the animal industry were less confident in interpreting veterinary referrals and gathering a clinical history from owners during an initial appointment. This was supported by interview data, where respondents also highlighted challenges with interpreting a veterinary referral early in their career. The findings of the present study are consistent with previous research in nurses and veterinary students, where communicating with patients was a challenge for recent nursing graduates^25^ and poor communication skills were identified as a concern by experienced veterinarians and students.^44^ Furthermore, training in communication skills in veterinary degrees and opportunities to practice after qualification were found to be significantly lacking,^45^, ^46^ which may also be true for current animal therapy qualifications. In addition, veterinarians reported that communication skills are developed primarily through real‐life patient interactions as opposed to training during veterinary degree programmes.^45^ However, improvements have recently been made in this area, with veterinary degrees introducing content on communication^47^ and CPD courses in communication being offered.^48^ Similar approaches can be considered for veterinary physiotherapy and qualifications could integrate approaches to teaching communication skills that have been investigated for human physiotherapy students, such as the use of e‐learning training packages.^49^

Confidence in recognising animals that required emergency care was another EPA that was impacted by experience in the animal industry. However, interview participants all mentioned that they very rarely encountered emergency situations, which potentially explains the lack of confidence of animal therapists in this area.

### The effect of experience as an animal therapist on confidence

While some EPAs were significantly impacted by experience as an animal therapist (interpreting a veterinary referral, recognising emergency situations), not as many were impacted by time spent in the therapist role as were affected by experience in the animal industry in general. Experience has previously been found to significantly impact confidence among several cohorts, including medical nurses^26^; however, this has not always been found to increase linearly^22^, ^23^, ^33^ with fluctuations in confidence being identified^22^, ^25^ based on the skills medical professionals are asked to perform. It has been identified that new veterinary graduates who lack experience rely heavily on colleagues for support.^50^ This is of relevance as veterinary physiotherapists are less likely to work as part of a wider team where they can benefit from the industry experience of their colleagues. It has been suggested that veterinary physiotherapists ‘shadow’ more experienced therapists to gain skills as part of their course,^43^ which is something that could potentially be required by governing bodies for new graduates.

### Approaches for improving confidence in animal therapists

The use of real‐life patients, an authentic work experience environment^36^ and the opportunity to access a diverse number of work experience placements^51^ were outlined as crucial factors in promoting professional development, confidence and preparedness to enter clinical practice in human physiotherapy students, suggesting that realism and immersivity could be essential in adequately preparing animal therapists for a clinical environment. Research in human physiotherapists has found the use of immersive simulations in teaching improves student confidence, which may be something that could be integrated into veterinary physiotherapy qualifications.^52^ In the present study, the sample size was small for the interviews, but interviewees commented on the lack of practical content in their qualifications, which is something that could be improved to further build confidence among animal therapists.

CPD is required to maintain skills and knowledge among human physiotherapists^53^ and is required by canine hydrotherapy and veterinary physiotherapy governing bodies; however, it is not a legal requirement at this time.^54^ This differs from the requirements for veterinarians who are mandated to complete CPD by the RCVS to enable them to practice in the UK.^55^ The number of different governing bodies in the field of animal therapy may result in individuals differing in the amount of CPD they complete. Therefore, a standardised approach to CPD might support animal therapists following the completion of their qualification. In the present study, interview respondents saw the benefit of CPD but cited lack of availability and cost as barriers to participation.

### Limitations and recommendations for future research

The interview participants demonstrated an awareness of their own limitations; however, it is unclear whether all participants were able to accurately assess their performance and provide realistic scores for EPAs. The Dunning‒Kruger effect may have affected the present study, as poor performers tend to overestimate their competency while high achievers tend to underestimate theirs.^56^ Previous literature has also outlined that personality traits may influence the development of professional confidence,^21^, ^22^ suggesting that some people may be unable to fully separate these concepts. Support from employers and mentors was found to be essential in a range of cohorts to increase confidence^25^, ^36^, ^57^, ^58^; therefore, determining whether an absence of this has an impact on confidence in animal therapists should be considered in future research. This is particularly relevant to the present study as the majority of the sample population worked in an independent business and may have lacked the support provided in a larger organisation. It would be interesting to compare the confidence of animal therapists who are self‐employed to that of those who work for a veterinary practice or larger organisation. In addition, why some therapists have taken the decision not to complete a qualification could be explored, as these unique insights may aid in the development of new therapy programmes. The sample population utilised for the interviews was quite small, meaning that it was not possible to extensively compare the experiences of more recent graduates to that of those who had worked in the industry for longer within this part of the dataset.

In conclusion, experience was an important determinant of the confidence of animal therapists in completing EPAs, with administrative EPAs particularly impacted by experience. Differences in confidence were also observed between some types of therapy qualification; hence, adjusting current courses may reduce these differences and ensure that all skills are equally developed. Standardising therapy qualifications and CPD requirements may help ensure that all therapists feel confident to enter clinical practice, thereby maintaining high standards of animal welfare.

## AUTHOR CONTRIBUTIONS

Both Alison Wills and Alice Sear contributed to the study design. Alice Sear administered the survey and interviews and analysed the data. Alison Wills and Alice Sear both contributed to the manuscript creation.

## CONFLICT OF INTEREST STATEMENT

This project did not receive any funding from any agency in the public, commercial or not‐for‐profit sectors.

## ETHICS STATEMENT

Ethical approval for this study was granted by Hartpury University Ethics Committee on 23 March 2022 (ETHICS2021‐78).

## Supporting information



Supporting information

## Data Availability

The data that support the findings of this study are available upon reasonable request by contacting the corresponding author.
