# Supplementary material for: UK animal therapists’ confidence in executing professional skills in clinical practice
Source: Vet Rec. 2024 Nov 17;195(11):e4704. doi: 10.1002/vetr.4704 (PMC11606001; doi:10.1002/vetr.4704)
Supplement: Supplementary file 1 — Supporting information [file VETR-195-e4704-s001.pdf]

Professional confidence in veterinary paraprofessionals

My name is Alice Sear, I am currently undertaking a Masters in Research degree at Hartpury University. I would like to invite you to take part in a project looking at professional confidence in animal hydrotherapists and veterinary physiotherapists based in the United Kingdom. The questionnaire should not take more than 10 minutes to complete.

The benefits of participation include the chance to contribute to a rapidly growing area of research.

Please note that all information which is collected about you during the course of the research will be kept securely with the researcher. Any information which enters the public domain will be anonymised so that you cannot be recognised from it unless you wish to be publicly acknowledged. Questionnaire data will be held but information will be coded for anonymity. The results will be presented as my dissertation and a paper and electronic copy will be kept at Hartpury University.

It is up to you to decide whether or not to take part in this research. By filling out this questionnaire this is indicating your voluntary informed consent to participating in the research and also granting consent for the data obtained from this to be used and analysed over the course of the research study.

If you decide to take part you are still free to withdraw without giving a reason up to when the designated point of withdrawal has been reached (data analysis) by contacting me, Alice Sear, on alice.sear@hartpury.ac.uk (mailto:alice.sear@hartpury.ac.uk).

* Required

1. Please outline a unique six-digit code which we can use to identify you, if you wish to withdraw from the study *

Demographics

2. Please state your age *

- ◯ Under 21
- ◯ 21-30
- ◯ 31-40
- ◯ 41-50
- ◯ 51-60
- ◯ 61-70
- ◯ 70+

3. Please specify your sex *

- ◯ Male
- ◯ Female
- ◯ Prefer not to say
- ◯ Not listed

4. Which of the following best describes your occupation? *

- ◯ Veterinary physiotherapist
- ◯ Veterinary physiotherapist and hydrotherapist
- ◯ Equine hydrotherapist
- ◯ Canine hydrotherapist

5. Which of the following species do you specialise in treating? *

- ◯ Companion animals including cats and dogs
- ◯ Small animals including guinea pigs and rabbits
- ◯ Equid species including horses and donkeys
- ◯ Large animals including cattle

6. Which of the following best describes your current working environment? *

- ◯ Primary care veterinary practice
- ◯ Referral veterinary practice
- ◯ Research or education facility
- ◯ Independent business

7. Approximately, how many years have you worked in the animal or veterinary industry including your time as an animal therapist? *

- ◯ Less than 1 year
- ◯ 1-5 years
- ◯ 6-10 years
- ◯ 11-20 years
- ◯ 21-30 years
- ◯ 31-40 years
- ◯ 41-50 years
- ◯ 51+ years

8. Approximately, how many years have you been in your current role as an animal therapist? *

- ◯ Less than 1 year
- ◯ 1-5 years
- ◯ 6-10 years
- ◯ 11-20 years
- ◯ 21+ years

9. What is your educational level? Please select the highest award you currently hold *

- ◯ None
- ◯ GCSE or equivalent
- ◯ A-Level or equivalent
- ◯ Bachelors degree
- ◯ Masters degree
- ◯ Doctorate

10. Please select the highest award you currently hold as an animal therapist? *

- ◯ None
- ◯ Level 2 certificate for equine hydrotherapy assistants
- ◯ Level 3 qualification in hydrotherapy for small animals
- ◯ Level 4 qualification in hydrotherapy for small animals
- ◯ Level 5 advanced certificate in hydrotherapy treatment techniques
- ◯ Level 6 diploma in veterinary physiotherapy
- ◯ Level 6 bachelors degree in veterinary physiotherapy
- ◯ Level 7 masters degree in veterinary physiotherapy with an undergraduate qualification
- ◯ Level 7 masters degree in veterinary physiotherapy with an undergraduate qualification in human physiotherapy

Professional confidence based on clinical skills

The following questions aim to determine professional confidence based on the principle of entrustable professional activities, which are essentially the most common skills encountered in clinical practice. Each is assorted in a random order and will be defined before you are asked to rank your agreement with the following statements in relation to confidence.

Self-assessed confidence can be subject to external influences, therefore remember to take your time to think and reflect on each scenario below before answering.

11. Gathering a clinical history during an initial appointment. This involves effectively communicating and asking specific questions to owners or caretakers regarding their animals health and wellbeing. How much do you agree with the statement below?

	Strongly disagree	Disagree	Neither disagree or agree	Agree	Strongly agree	Not applicable
I am able to gather a clinical history during an initial appointment	○	○	○	○	○	○

12. Performing a routine physical examination. This involves observing and palpating the patient in an appropriate manner to determine general health status and specific considerations relating to the nature of the condition. How much do you agree with the statement below?

	Strongly disagree	Disagree	Neither disagree nor agree	Agree	Strongly agree	Not applicable
I am able to perform a routine physical examination of the patient	○	○	○	○	○	○

13. Interpreting a veterinary referral. This involves understanding the terminology utilised by veterinary surgeons to understand the nature and severity of the condition, in addition to the purpose of treatment. How much do you agree with the statement below?

	Strongly disagreee	Disagree	Neither disagree nor agree	Agree	Strongly agree	Not applicable
I am able to interpret a veterinary referral	○	○	○	○	○	○

14. Interpreting a clinical history. This involves understanding patient records by deciphering terminology and veterinary jargon to gain a picture of the patients' general health, in addition to specific details regarding the nature of the condition. How much do you agree with the statement below?

	Strongly disagree	Disagree	Neither disagree nor agree	Agree	Strongly agree	Not applicable
I am able to interpret a clinical history	○	○	○	○	○	○

15. Developing and implementing a management or treatment plan for a patient. This involves utilising referral and clinical history documents, in addition to the clinical examination to set realistic goals for treatment. This also includes the ability to adjust the treatment plan when necessary. How much do you agree with the statement below?

	Strongly disagree	Disagree	Neither disagree nor agree	Agree	Strongly agree	Not applicable
I am able to develop and implement a management or treatment plan for a patient	○	○	○	○	○	○

16. Carrying out a dynamic risk assessment. This involves identifying and reacting to potential hazards in the workspace to protect yourself, the general public, patients and colleagues from harm. How much do you agree with the statement below?

	Strongly disagree	Disagree	Neither disagree nor agree	Agree	Strongly agree	Not applicable
I am able to carry out a dynamic risk assessment	○	○	○	○	○	○

17. Recognising a patient requiring urgent or emergency care and initiating the appropriate action. This involves knowledge of potential emergency situations and the ability to apply this knowledge under pressure. This also involves the ability to judge ones limitations and to seek help and immediate assistance. How much do you agree with the statement below?

	Strongly disagree	Disagree	Neither disagree nor agree	Agree	Strongly agree	Not applicable
I am able to recognise a patient requiring urgent or emergency care and initiate the appropriate action	○	○	○	○	○	○

18. Communicating with veterinary personnel such as receptionists, veterinary nurses and other veterinary paraprofessionals. This involves the ability to give, receive and interpret information through written and verbal means relating to a patients treatment. How much do you agree with the statement below?

	Strongly disagree	Disagree	Neither disagree nor agree	Agree	Strongly agree	Not applicable
I am able to communicate with veterinary personnel such as receptionists, veterinary nurses or other veterinary paraprofessionals	○	○	○	○	○	○

19. Communicating with veterinary surgeons. This involves the ability to give, receive and interpret information through written and verbal means relating to a patients treatment. How much do you agree with the statement below?

	Strongly disagree	Disagree	Neither disagree nor agree	Agree	Strongly agree	Not applicable
I am able to communicate with veterinary surgeons	○	○	○	○	○	○

20. Keeping accurate records including the content of each treatment session. This involves recording specific details of each patients session which can be easily interpreted by a person with limited prior knowledge of the patient. How much do you agree with the statement below?

	Strongly disagree	Disagree	Neither disagree nor agree	Agree	Strongly agree	Not applicable
I am able to keep accurate records including the content of each treatment session	○	○	○	○	○	○

21. Applying new techniques or knowledge gained from CPD courses or equivalent into clinical practice. This involves understanding the appropriateness of new techniques which aim to enhance treatment sessions, in addition to the application of these techniques without supervision. How much do you agree with the statement below?

	Strongly disagree	Disagree	Neither disagree not agree	Agree	Strongly agree	Not applicable
I am able to apply new techniques or knowledge gained from CPD or equivalent into clinical practice	○	○	○	○	○	○

22. Thank you for your participation!

If you would be interested in being contacted for an interview regarding your experiences with professional confidence in clinical practice, please provide an email address below:

23. If you have been affected by the issues raised within this survey please utilise the link outlined below which will direct you to contacts recommended by the mental health based charity Mind.

https://www.mind.org.uk/information-support/types-of-mental-health-problems/anxiety-and-panic-attacks/useful-contacts/ (https://www.mind.org.uk/information-support/types-of-mental-health-problems/anxiety-and-panic-attacks/useful-contacts/)

This content is neither created nor endorsed by Microsoft. The data you submit will be sent to the form owner.

Microsoft Forms
